# Acute effects of aerobic exercise promote learning

**DOI:** 10.1038/srep25440

**Published:** 2016-05-05

**Authors:** Renza Perini, Marta Bortoletto, Michela Capogrosso, Anna Fertonani, Carlo Miniussi

**Affiliations:** 1 Department of Clinical and Experimental Sciences, University of Brescia, Viale Europa 11, 25123 Brescia, Italy; 2 Cognitive Neuroscience Section, IRCCS Centro San Giovanni di Dio Fatebenefratelli, Via Pilastroni 4, 25125 Brescia, Italy

## Abstract

The benefits that physical exercise confers on cardiovascular health are well known, whereas the notion that physical exercise can also improve cognitive performance has only recently begun to be explored and has thus far yielded only controversial results. In the present study, we used a sample of young male subjects to test the effects that a single bout of aerobic exercise has on learning. Two tasks were run: the first was an orientation discrimination task involving the primary visual cortex, and the second was a simple thumb abduction motor task that relies on the primary motor cortex. Forty-four and forty volunteers participated in the first and second experiments, respectively. We found that a single bout of aerobic exercise can significantly facilitate learning mechanisms within visual and motor domains and that these positive effects can persist for at least 30 minutes following exercise. This finding suggests that physical activity, at least of moderate intensity, might promote brain plasticity. By combining physical activity–induced plasticity with specific cognitive training–induced plasticity, we favour a gradual up-regulation of a functional network due to a steady increase in synaptic strength, promoting associative Hebbian-like plasticity.

How often have we heard, “Mens sana in corpore sano”, i.e., “a sound mind in a sound body”, which suggests that only a healthy body can sustain a healthy mind. Nevertheless, although this adage has been widely used for some time, its foundational notions must still be substantiated. While the benefits that physical activity confers on cardiovascular health are well known, the idea that exercise can also increase brain “performance” has only recently begun to be investigated by neuroscientists. Thus, whether and how physical exercise makes us cognitively more resourceful has been only partially explored.

Several recent studies have shown that regular aerobic physical exercise might improve cognitive functions by helping functional recovery after brain injury and by preventing cognitive decline in normal ageing (for a review see^1^). Moreover, many observational studies have noted good cognitive performance in subjects who report practicing regularly physical activity^2^,^3^. Consistent with these observations, there are also structural imaging studies confirming an association between physical activity and increased grey matter volume in subjects that exercise regularly in comparison with sedentary people^4^,^5^,^6^. Nevertheless, some of these studies have been criticized because of the presence of other direct causal links between physical activity and cognitive performance; for instance, high cognitive abilities are more likely to be associated with higher educational levels, which are, in turn, often associated with a more health-conscious life style.

To overcome these problems, other studies have concentrated on the benefits of the acute effects of physical activity on cognitive processing, irrespective of the previous fitness of tested subjects. These studies compared the subjects’ cognitive performance immediately before and after a single bout of aerobic exercise (for a review see^7^), and some found an improvement in attention, visuospatial functions, memory, language and executive functions e.g.^2^,^8^,^9^,^10^,^11^. However, many studies have reported no significant improvement in cognitive performance after physical activity, as shown in a recent review of more than 30 studies^12^.

Evidence from animal studies suggest that neurotrophic factors (i.e., brain-derived neurotrophic factor - BDNF) might play a key role in such effects^13^,^14^, and this evidence has also been confirmed in research on humans^15^,^16^,^17^. These studies have shown a relevant and constant increase of BDNF concentration up to 60 minutes following aerobic exercise.

Specific work on BDNF has shown that this factor plays a pivotal role in the induction of activity-dependent neuroplasticity^18^. Thus, it can be inferred that the advantage of physical exercise may involve directly affecting synaptic plasticity by favouring the strengthening of network structures, supporting neurogenesis and favouring metabolism and vascular functions^19^. Accordingly, a few studies have shown that a single bout of physical exercise enhances neuroplasticity^20^,^21^. As a consequence, physical exercise might have a direct effect on the enhancement of brain functioning. Regular participation in physical activity has been associated with improved cognitive functions across the lifespan^22^,^23^. Moreover, it may help prevent and even treat cognitive decline, thereby promoting general health. Given this promising potential, such an area of research is important not only to understanding the mechanisms that might lead to improved cognition in healthy subjects but also to discovering novel strategies targeting neurorehabilitation and pathological ageing in an ageing society.

In the present study, we used a sample of young male subjects to test the effects of a single bout of aerobic exercise on learning. Although previous studies have shown that physical activity can enhance cognitive performance and alter motor cortex excitability, we investigated whether such an enhancement is also present at a learning level within visual and motor domains (with only the latter directly associated with the physical activity). Therefore, the distinctive contribution of the present study is that both visual and motor domains have been tested with the same approach, i.e., with learning tasks that involve changes in synaptic activity. Importantly, the chosen learning tasks are considered to reflect a direct manifestation of neural plasticity^24^,^25^,^26^. Finally, beside measuring the impact of physical exercise on general overall performance, we investigated if this effect was associated with a change in learning rate.

First, we employed an orientation discrimination task (ODT)^27^, which is a type of perceptual learning task and involves changes in the synapses within the primary visual cortex (V1). Second, we adopted a simple thumb abduction motor task (MT)^28^ in which learning relies on changes in the activity of the primary motor cortex (M1). We hypothesized that if a single session of aerobic exercise increased the release of neurotrophic factors concentration, as previously suggested, then such an increase should be available to substantially enhance plasticity in all areas of the brain that are specifically engaged in activity-dependent learning – learning that should result in task-performance improvement. Thus, our study on learning goes beyond the effects of a more general cognitive performance and may increase our understanding regarding how physical activity may trigger specific plasticity mechanisms in healthy adults.

## Materials and Methods

The present study was approved by the Ethics Committee of the IRCCS San Giovanni di Dio Fatebenefratelli, Brescia and was conducted in accordance with the Declaration of Helsinki. Written informed consent was obtained from all participants prior to the beginning of the experiment.

### Subjects

Healthy male participants with no heart problems and no neurological or psychiatric disease participated in the study. All participants were university students, right-handed with normal or corrected-to-normal vision. They were randomly assigned to the exercise or control group (see Experimental Procedure).

Forty-four volunteers participated in Experiment 1 [age (mean ± st.dev): 23.0 y ± 2.2; weight: 75.6 Kg ± 10.7; height: 1.79 m ± 0.07; BMI: 23.5 ± 2.8]. Data from six participants were excluded either because the subjects did not reach an established learning performance in the task (four subjects), or because their performance was more than two standard deviations from the mean (two subjects). On the basis of these criteria, the aerobic exercise group and control group included 18 and 20 participants, respectively (age: 23.0 y ± 2.5; weight: 76.0 Kg ± 11.2; height: 1.79 m ± 0.06; Body Max Index-BMI: 23.7 ± 3.0).

Forty volunteers participated in Experiment 2 (age: 22.9 y ± 2.2; weight: 76.2 Kg ± 10.8; height: 1.80 m ± 0.06; BMI: 23.6 ± 2.9). Data obtained from three participants were excluded either because of technical problems during data acquisition (two subjects), or because their performance was more than two standard deviations from the mean (one subject). On the basis of these criteria, the aerobic exercise group and control group included 18 and 19 participants, respectively (age: 22.9 y ± 2.4; weight: 75.9 Kg ± 11.1; height: 1.79 m ± 0.06; BMI: 23.6 ± 3.0). Thirty-three of the volunteers participated in both Experiment 1 and Experiment 2. We chose to test the same subjects (as much as possible) to reduce variance associated with individual differences. Additionally, only males were tested because we did not want to introduce a possible additional confounding factor related to the hormonal status. In this respect, an analysis by Colcombe and Kramer^29^ revealed that when participant sample included more than half females, the group as a whole showed greater benefit from physical activity than if the sample included less than half females. So while exercise induces benefits for both genders, it may be of greater benefit to female.

## Experimental Procedure

### Physical activity

In the first session, all the participants were requested to complete both a health history and International Physical Activity Questionnaire (IPAQ, Short Last 7 days Form; www.ipaq.ki.se) to assess the individual level of physical activity participation^30^. After completing these questionnaires, anthropometric (height, body weight) measurements, heart rate (HR) and blood pressure (BP) were assessed at rest in a sitting position. Next, the participants performed a submaximal cycle test, following the YMCA protocol to indirectly estimate maximal oxygen consumption (VO2max)^31^.

**Figure 1:**
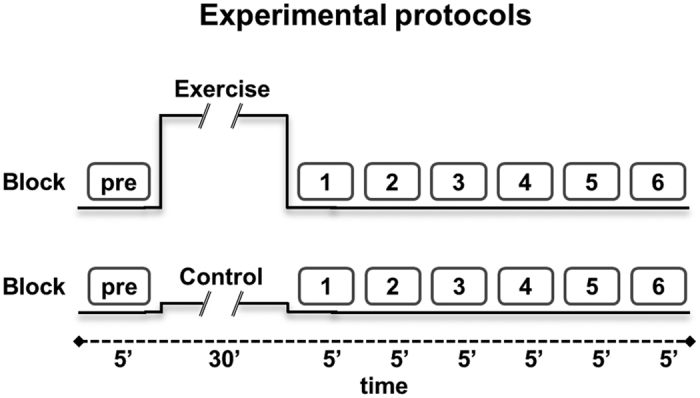
Experimental protocol for exercise (70% HRmax) and control (20 W) conditions. One block (5 minutes) of the task (ODT or MT) was performed before the cycle-pedalling session (exercise or control), and six blocks (corresponding to 30 minutes) were performed thereafter. HR was continuously recorded during the entire experimental session.

In a second session, the participants performed the experimental protocol depicted in Fig. 1. In both Experiment 1 and Experiment 2 (see below for details), the participants sat on the cycle after performing the first block (pre) of learning task, rested for some minutes, and then pedalled at 60 revolutions per minute for 30 min. The exercise group was requested to work at 70% of HRmax, which corresponded to 157 ± 44 W in Experiment 1 and 159 ± 47 W in Experiment 2. The control group pedalled at 20 W, i.e., at a load eliciting a very slight increase in metabolic rate. Immediately after the end of the exercise, the participants performed additional six blocks of learning tasks in approximately 30 min.

HR was continuously recorded throughout the experiment using the Polar HR monitor (Polar RS800CX, Polar Electro, Kempele, Finland). HR was then averaged on a 2 min basis during exercise and over the entire duration of each block (HR block-n). Ratings of perceived exertion (RPE) were evaluated during pedalling, using the Borg scale (6–20) at 10 min intervals.

### Experiment 1 - Orientation discrimination task (ODT)

Participants were seated in front of a computer screen in a quiet, semi-dark room, with their chin placed on a chin rest 57 cm from the screen. The subjects were presented visual stimuli in couples (black lines, 2° long and 5′ wide in visual angle) and were asked to determine whether the stimulus presented second was tilted clockwise or counter clockwise relative to the stimulus presented first. To limit the area in which they were presented, a piece of black cardboard covered the screen except for a 10 cm diameter circle in the centre of the screen. Stimuli were presented in each of the four visual hemi fields: upper left and upper right, lower left and lower right (Presentation software v. 12.0). In each trial, the two lines were presented in the same hemifield. The orientation of the reference stimulus was 45° in the upper right and lower left hemi fields and 135° in the upper left and lower right hemi fields. The angular differences between the reference and target stimulus were 1.1, 1.21, 1.33 and 1.46°. The reference was presented first in half of the trials and second in the other half of the trials. All previously described experimental parameters were balanced and randomized between blocks. A central fixation point was maintained for the duration of the trial.

**Figure 2:**
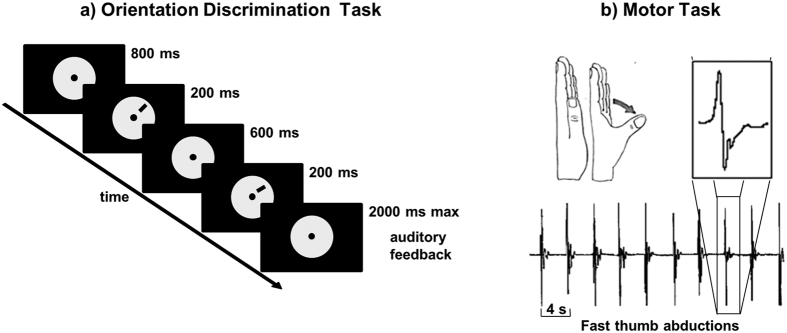
Trial structure. (**a**) An example of an orientation-discrimination task trial with the reference and target stimuli presented in the upper right hemifield. The line can turn clockwise or counter-clockwise. The subjects were asked to determine whether the presented stimulus was tilted clockwise or counter-clockwise relative to the previously presented stimulus. (**b**) The motor task consisted of rapid thumb abduction movements of the left hand, which induced learning. Movement accelerations were measured during the entire experiment and are represented in the lower part of the figure and amplified in the right upper part.

The participants were asked to respond as quickly and accurately as possible after the second stimulus was presented by pressing the left (counter clockwise) or right (clockwise) button of a response pad with the left or right index finger, respectively. Auditory feedback (duration = 50 ms; frequency for a correct response = 700 Hz; frequency for an incorrect response = 350 Hz) informed the subjects about the correctness of their responses. The trial structure is described in Fig. 2a.

During the experimental session, the participants performed an initial training block (consisting of only 8 trials), followed by 7 experimental blocks. One experimental block was run before the exercise as baseline (pre) and six after the exercise (post). Each block consisted of 64 trials and lasted approximately 4 minutes. Blocks were performed with 40 sec breaks between blocks for a total duration of approximately 30 minutes. We chose the ODT because it is a visual-perceptual task that implies learning and plasticity at the level of the V1 cortex^27^,^32^,^33^.

### Data Analyses: ODT

The average orientation sensitivity was calculated in terms of the *d’* value for each subject and each block. The *d’* is a measure of sensitivity of detection theory and it is calculated as the difference between the z transformation of hit rate and false alarm rate. Because the ODT is a two-alternative forced-choice task, the *d’* was divided by square root of two so that *d’* = 1 corresponded to 75% accuracy^34^. The *d’* values were referenced to the baseline (Δ*d’* = *d’* block n − *d’* baseline) by calculating the difference (block-baseline). As an index of learning rate, we analysed the relationship between *d’* and the block number using linear regression analysis. We obtained a slope value for each subject and then we compared the slopes between exercise vs. control group with a one-way ANOVA.

A Kolmogorov-Smirnov test confirmed the normality of the distribution for all data (*d’*, slopes). A mixed-design ANOVA was performed, including physical activity (exercise vs. control group) as a between-subject factor and block (from 1 to 6) as a within-subject factor.

Data sphericity was tested using the Mauchly test where appropriate, applying the Greenhouse–Geisser correction when appropriate and Fisher’s least significant difference method for post-hoc tests. A *p*-value < 0.05 was considered significant for all statistical analyses. We tested the effects of physical exercise on the learning rate by comparing the slopes from the linear regression analyses of the exercise group and the control group.

### Experiment 2 - Motor task

Participants were comfortably seated on an armchair, looking at a computer screen placed in front of them. Their left arm was abducted at the shoulder and flexed 90° at the elbow, and the semi pronated forearm rested on a flat armrest. The forearm, wrist and fingers II–V were fixed in a cast, leaving the thumb free for horizontal movements.

The MT consisted of repeatedly performing abduction movements with the left thumb as fast as possible. These movements are known to induce learning and plasticity in M1^25^,^28^. Participants performed blocks of 65 movements paced by a brief 1000 Hz tone at a rate of 0.25 Hz while receiving verbal encouragement from the experimenter and visual feedback of the movement acceleration. Before each movement, the thumb had to be relaxed into a resting position, leaning against a soft support that corresponded to the starting position. The use of the support prevented shifting the thumb’s starting position during practice as a potential cause for performance change. The trial structure is represented in Fig. 2b.

The task was performed in blocks as for the ODT task: 1 block (approximately 5 min) before the exercise and 6 blocks after the exercise (30 min with 40 sec breaks). Before the task, the participants practiced the movement 10 times. Movement acceleration was measured during the entire experiment using a monoaxial accelerometer (model: 352C23, voltage sensitivity = 5 mV/g; PCB piezotronic) mounted on the thumb’s interphalangeal joint to detect acceleration along the abduction–adduction axis. The raw signal was amplified with a gain factor of 100 (model: 480E09 signal conditioner; PCB piezotronic), digitized (A/D rate: 500 Hz) and fed into the laboratory computer for online visual display and offline analysis.

### Data Analyses: MT

Movement acceleration (Acc) was measured at the first peak of the accelerometer signal and was baseline-corrected to the 100 ms preceding the beginning of the movement. The mean Acc was calculated for each block. Next, the values of Acc after physical exercise were referenced to the baseline (ΔAcc = Acc block n − Acc baseline) by calculating the difference (block-baseline).

With respect to the ODT, we performed two analyses. First, a mixed-design ANOVA was performed, including physical activity (exercise vs. control group) as a between-subject factor and block (from 1 to 6) as a within-subject factor, applying the Greenhouse–Geisser correction when appropriate and Fisher’s least significant difference method for post-hoc tests. A p-value < 0.05 was considered significant for all statistical analyses. Second, we tested the effect of physical exercise on the learning rate by comparing the slopes from the linear regression analyses of the exercise group and the control group.

## Results

### Physical activity

On the basis of the reported frequency and duration of the different forms of physical activities requested by IPAQ, the level of physical activity resulted “moderate” or “high”. Only three subjects were categorized as “low”, i.e., not reaching 600 MET-min/week^30^.

No differences were found in baseline HR, systolic BP and diastolic BP between the two groups in both Experiment 1 and Experiment 2: their mean values are reported in Table 1 for the exercise and control groups together with VO2max (*p* > 0.05 between groups).

In Experiment 1, the HR in the last 2 minutes of pedalling (exercise HR in Table 1) increased above the resting rate of approximately 80 b/min and 4 b/min, respectively in the exercise and control groups (p < 0.05 between groups). Similar increments were observed at the end of pedalling in Experiment 2, namely 75 b/min in the exercise group and 7 b/min in the control group. The RPE values of the exercise group were similar in Experiment 1 and Experiment 2; in particular, in the last part of the task, it was between 13 and 15 (somewhat hard and hard). However, the control group reported an RPE score corresponding to very, very light or very light.

In both experiments, in the initial 2 min of recovery after task at 70% HRmax, HR decreased by approximately 50 b/min, and then further slowly decreased to attain rest values after approximately 20 min. However, after the end of pedalling at 20 W, HR demonstrated a tendency to slightly decrease below rest values.

During the initial block (pre-exercise), similar HRs were observed independent of group and experiment and were no different from rest values. During the blocks after pedalling, a continuous decrease in HR occurred in the exercise group from a value of 99 b/min and 90 b/min in block-1 to 77 b/min and 79 b/min in block-6 in Experiments 1 and 2, respectively (see Table 1). In the control group, HR was constant during all blocks in both experiments (approximately 62 b/min and 67 b/min in Experiments 1 and 2, respectively).

### ODT

Orientation sensitivity – Δ*d’*: The repeated measures ANOVA with block as a within-subjects factor and physical activity as between-subjects factors highlighted a significant main effect for block [F(5,180) = 5.29; *p* < 0.01]. Moreover, the interaction between physical activity (exercise vs. control group) and block (1–6) was statistically significant [F(5,180) = 2.26; *p* < 0.05]. In the exercise group, orientation sensitivity showed a continuous increase in successive blocks (1 ≠ 4, 5, 6; 2 ≠ 4, 5, 6; 3 ≠ 6). By contrast, in the control group, no modification in *d’* value occurred from block 1 to block 6 (only block 4 ≠ 5, 6).

**Figure 3:**
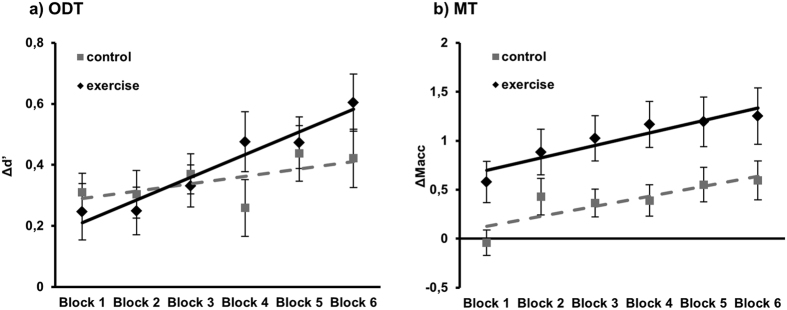
Experimental results. Data are represented as the mean ± MSE. The lines represent the fit of each condition: The black solid line (diamonds) represents the exercise condition, and the grey dashed line (squares) represents the control condition. Blocks 1–6 refer to the blocks of the tasks performed after the physical exercise. (**a**) Results of Experiment 1 (ODT). For each condition, data are presented as the difference between mean *d’* values of each block and the baseline. (**b**) Results of Experiment 2 (MT). For each condition, data are presented as the difference between movement acceleration of each block and the baseline.

To specifically evaluate learning rate differences, we compared learning performance in the two conditions after the physical activity. In this analysis, stimulation condition results were significant [F(1, 36) = 4.65; *p* = 0.037], which suggested the presence of a different learning rate between the two groups: the results indicated a lower learning rate in the control group than in the exercise group (See Fig. 3a).

### MT

We observed motor learning and performance improvement in the MT [Main effect block: F(2.30, 80.64) = 8.17, ε = 0.46, *p* < 0.001]. In the post-hoc analyses, Acc was smaller in the first block than in all the other blocks, smaller in the second block than in the last block, and smaller in the third block than in the last block (all *p* < 0.05), showing that Acc increased with practice of the MT.

Importantly, performance of the MT was affected by the execution of physical activity [Main effect physical activity: F(1, 35) = 6.37, *p* < 0.05]. Specifically, Acc was higher overall for the exercise group in comparison with the control group.

No significant interaction was found between the factors of physical activity and block (*p* = 0.85). Similarly, the learning rate after physical exercise did not change between groups, as shown by the non-significant effect on the slope (*p* = 0.71) (See Fig. 3b).

## Discussion

In this study, we demonstrated that aerobic exercise can enhance the ability of young male adults to learn within visual and motor domains and that these positive effects can persist after the exercise is over. Specifically, a single bout of aerobic exercise can enhance a basic learning process, irrespective of the involved cortex (visual or motor) for at least 30 minutes following termination of the exercise. In Experiment 1, we evaluated the subjects’ performance in a perceptual learning task – a form of implicit memory characterized by improvement in sensory discrimination after repeated exposure to a particular type of stimuli. The underlying neural plasticity involves changes in the responsiveness of neurons in the V1 area^35^ in relation to learning and memory processes. In Experiment 2, we evaluated the subjects’ performance in a simple motor task characterized by rapid ballistic movements. Such elementary motor behaviour induces learning associated with increased excitability of M1, indicating that human M1 is specifically engaged during the early stages of motor memory^28^. Both of these forms of learning are considered a direct manifestation of neural plasticity and are thus directly influenced by factors implied in the neuroplasticity process.

Previous studies have shown that one of the mechanisms that mediate the effects of exercise is an increased concentration of BDNF secretion in the brain (e.g.^2^,^13^,^16^,^36^; for a review see^17^). Given these premises, we speculate that if a single session of aerobic exercise augmented the release of neurotrophic factors that facilitate neuroplasticity, then the synapses that are active may be those that are mainly advantaged. Indeed, BDNF supports the survival of existing neurons, and encourage the growth and differentiation of new neurons and synapses in the whole nervous system^37^. Thus, an increase in BDNF will be available to the brain areas that are active and will upregulate their activity, favouring activity-dependent learning. Consequently, we expected to observe an effect of physical activity mainly on a specific network linked to task performance, the one activated with these tasks (i.e., those performed by the subjects), because the synapses of these networks should be those profiting more from changes due to the presence of neurotrophic factors.

In this context, an important question is related to the dimension of the advantage induced by physical activity in relation to the cognitive tests evaluated. Cognitive functions rely on the activity of brain circuits, and the synapses of such circuitry must be activated to exploit the increased “plasticity” induced by physical activity, if this is the mechanism. In this respect, one factor to be considered is that the majority of the studies that reported lack of improvement investigated functions that imply complex cognitive circuits, and these functions were evaluated without specific learning interventions. Consequently, some studies might have underestimated the physical exercise–related effects on neuro-cognitive performance because such functions must take advantage of the neuroplasticity increase offered by “physical exercise” through direct activation. Cognitive improvement in this context should be considered a form of synaptic activity through co-activation by physical exercise and task, inducing an activity-dependent modification on a specific network(s). If such co-activation does not occur because the task is not performed in a manner that potentiates specific connectivity, then no advantage would be observed.

It has been suggested that different forms of physical training affect different neuro-cognitive networks^3^, likely depending on the network engaged to perform such specific training. Consistent with such a notion, it has been demonstrated that functions showing more intra-subject variability and/or undergoing more changes across the life span should profit more from physical activity^3^. Because these functions are the most sensitive to changes in synaptic connectivity, they are more likely to obtain benefits from substances that promote neuroplasticity. Thus, one necessary component when cognitive function is tested after physical activity is to train these functions in the experimental and control condition to establish whether there are advantages conferred only on the experimental condition.

We found that a single session of aerobic exercise facilitated basic learning mechanisms, suggesting that physical activity – at least of moderate intensity – could promote brain plasticity in young male subjects in motor and non-motor areas. Previous work from Fabre *et al*.^38^ showed that a combination of physical and cognitive training might be more successful in improving cognition than either intervention alone^39^,^40^. Furthermore, recent findings have shown that a single session of physical exercise increases functional connectivity in specific brain networks^41^,^42^. This result underlies the importance of the network that is “on duty” after the physical activity. Indeed, an increase in connectivity is one of the important keys to understanding the functional effects induced by exercise at the brain level.

These findings are important not only for cognitive functions but also for motor functions that are often the primary goal of neurorehabilitation strategies^43^. Exercise has a significant action on brain function, affecting fundamental aspects of brain plasticity^20^,^21^,^44^; to broaden such actions, the combination of training sessions (physical and cognitive) might be an optimal approach alone^39^,^40^. By combining physical activity–induced plasticity with specific cognitive training–induced plasticity, we might favour a gradual up-regulation of a functional network due to the steady increase in synaptic strength, which would promote associative, Hebbian-like, plasticity. Such approach can be defined as network-activity-dependent intervention, given that the neuronal activation that is induced by physical activity would prime the nervous system^21^ favouring the efficacy of specific functional training.

The benefits to physical health that arise with aerobic exercise are many and well-documented and include cardio-respiratory fitness, muscle oxidative capacity, decreased adiposity and associated body-mass index, glucose and lipid homeostasis, metabolic health, inflammatory burden, muscle mass and strength increase and reduction in mortality risk e.g.^45^,^46^,^47^. These aspects are important in a society in which the social and economic costs of caring for “unhealthy adults” are enormous. Importantly, we have shown that the benefits of physical activity can be extended also to “cerebral health” and can thus be exploited in the neuropathological ageing (e.g., cognitive impairment and dementia) and other brain disorders. The goal of future research in this field should be the maintenance or improvement and restoration of “cerebral health”. We propose that this objective can be reached with approaches involving brain plasticity induction to re-balance, compensate and control the changes in cortical connectivity.

## Additional Information

**How to cite this article**: Perini, R. *et al*. Acute effects of aerobic exercise promote learning. *Sci. Rep*. **6**, 25440; doi: 10.1038/srep25440 (2016).

## Figures and Tables

**Table 1:** Physiological and behavioural results in the baseline physiologic measurements (first session), in Experiment 1 and in Experiment 2.

	Exercise group (70% HR max)	Control group (20 W)
**Baseline physiological measurements**		
HR (b/min)	72.5 ± 12.1	75.8 ± 12.4
SBP (mmHg)	120.2 ± 9.2	117.1 ± 11.1
DBP(mmHg)	82.2 ± 8.9	78.9 ± 11.7
VO2max (ml/min/kg)	51.4 ± 5.6	50.7 ± 6.6
**Experiment 1**		
**ODT (Δd’ ± SD)**		
Block1	0.25 ± 0.39	0.31 ± 0.28
Block2	0.25 ± 0.33	0.30 ± 0.35
Block3	0.33 ± 0.29	0.37 ± 0.30
Block4	0.48 ± 0.42	0.26 ± 0.41
Block5	0.47 ± 0.36	0.44 ± 0.41
Block6	0.60 ± 0.40	0.42 ± 0.43
**HR (b/min)**		
Resting	72.8 ± 12.3	76.3 ± 9.3
Exercise	153.6 ± 12.2	79.9 ± 15.0
Block1	98.9 ± 12.9	63.4 ± 10.7
Block6	77.8 ± 10.7	60.9 ± 9.6
**Experiment 2**		
**MT (ΔACC ± SD)**		
Block1	0.58 ± 0.90	−0.04 ± 0.57
Block2	0.89 ± 0.99	0.43 ± 0.81
Block3	1.03 ± 0.98	0.36 ± 0.62
Block4	1.17 ± 1.00	0.39 ± 0.70
Block5	1.19 ± 1.08	0.55 ± 0.77
Block6	1.25 ± 1.22	0.60 ± 0.86
**HR (b/min)**		
Resting	73.1 ± 12.3	75.9 ± 9.5
Exercise	148.0 ± 13.4	83.5 ± 12.8
Block1	89.3 ± 10.3	67.9 ± 13.4
Block6	78.7 ± 9.33	68.3 ± 41.4

HR: heart rate; BP: blood pressure; ODT: orientation discrimination task; ACC: movement acceleration; resting: sitting on the cycle; exercise: last 2 min of pedalling. Behavioural data are reported as Δ (difference between mean values of each block and the baseline) ± standard deviation.
